# Combined Therapy for Associated Orofacial Disorders—A Challenging Case Report

**DOI:** 10.1055/s-0042-1749365

**Published:** 2022-07-12

**Authors:** Denise Fernandes Barbosa, Laura Fernandes Bana, Fausto Berzin, Almiro José Machado Júnior

**Affiliations:** 1 Division of Surgical Sciences, Department of Otorhinolaryngology, School of Medical Sciences, University of Campinas, UNICAMP, Campinas, São Paulo, Brazil; 2 Department of Morphology, Dentistry School of Piracicaba, Piracicaba, São Paulo, Brazil

**Keywords:** bruxism, neck pain, occlusal splint, occlusal adjustment, temporomandibular joint dysfunction, tinnitus

## Abstract

Health promotion and disease prevention link intricately with lifestyle habits such as a healthy diet, physical activity, and good sleep quality. Temporomandibular joint (TMJ) dysfunction and associated disorders can take away sleep and well-being depending on the form and intensity that affect the individual. A multidisciplinary effort has contributed to significant health advances, improving clinical outcomes concerning TMJ dysfunction. This report presents the case of a 37-year-old Caucasian female physical educator with a good healthy diet with complaints of tooth tightening, constant TMJ and neck pain, and tinnitus. The patient was treated with inferior occlusal splint placement and selective occlusal adjustments based on neuro-occlusal rehabilitation. The patient reported relief of pain symptoms with occlusal and body balance, discontinued analgesic medication, and maintained the occlusal splint to practice sports and sleep due to the perception of improved physical performance and sleep, and quality of life. Based on this report, it is necessary to analyze the causes and define the effects of different disorders to establish their diagnosis and treatment and changing patterns to reestablish functional balance.

## Introduction

Health promotion and disease prevention link intricately with lifestyle habits such as a healthy diet, physical activity, and good sleep quality. Temporomandibular joint (TMJ) dysfunction and associated disorders can take away sleep and well-being depending on the form and intensity that affect the individual. A multidisciplinary effort has contributed to significant health advances, improving clinical outcomes concerning TMJ dysfunctions. 1

This study aimed to report a challenging case of TMJ dysfunction associated with tinnitus, 2 cervicalgia, 3 and bruxism 4 treated and controlled with an analogic occlusal splint 5 and selective occlusal adjustments. This treatment is based on the neuro-occlusal rehabilitation (NOR) proposed by Pedro Planas laws 6 approach for improving mandibular movements with the functional balance 7 of the stomatognathic system respecting the minimum vertical dimension. 8

## Case Report

A 37-year-old Caucasian female patient, body mass index of 21.60 kg/m ^2^ , a physical educator with a good healthy diet presented with tooth tightening, constant TMJ and neck pain, and tinnitus. During anamnesis, the patient complained about poor sleep quality and impairment of daily activities due to TMJ pain and tinnitus, which were not relieved by taking analgesic medication. All pain complaints presented on the right side. These complaints were associated with tension and fatigue in the temporal and masseter region, mouth opening limitation, difficulty in chewing hard foods, pain and edema around the neck, and vicious habit of tooth tightening with the sensation of upper teeth moving forward.

The patient sought dental care for the first time to treat these problems. In clinical examination, we observed angle class I occlusal key with tooth biprotrusion, mouth opening limitation (2 cm), and severe occlusal wear sign only on the left side second molars. In physical examination, all pain symptoms related to muscles palpation on the right side: deep part masseter, anterior temporal, trapeze, and sternocleidomastoid. Articular TMJ presents no dysfunctional clinic signs. The initial complaint on the visual analog scale (VAS) was 6/7.

After anamnesis, clinical, and physical examination using the diagnostic criteria for TMJ dysfunction, the patient was diagnosed with muscular TMJ dysfunction—axis I with chronic facial pain and axis II with pain-related impairment of daily activities. The TMJ dysfunction is most likely due to psychosocial character, exacerbated by bruxism in sleep and wakefulness. Additionally, occlusal trauma is a risk factor for pain associated with cervicalgia and tinnitus. The tinnitus is due to probable retrodiscal zone compression and inflammation.

**Fig. 1:**
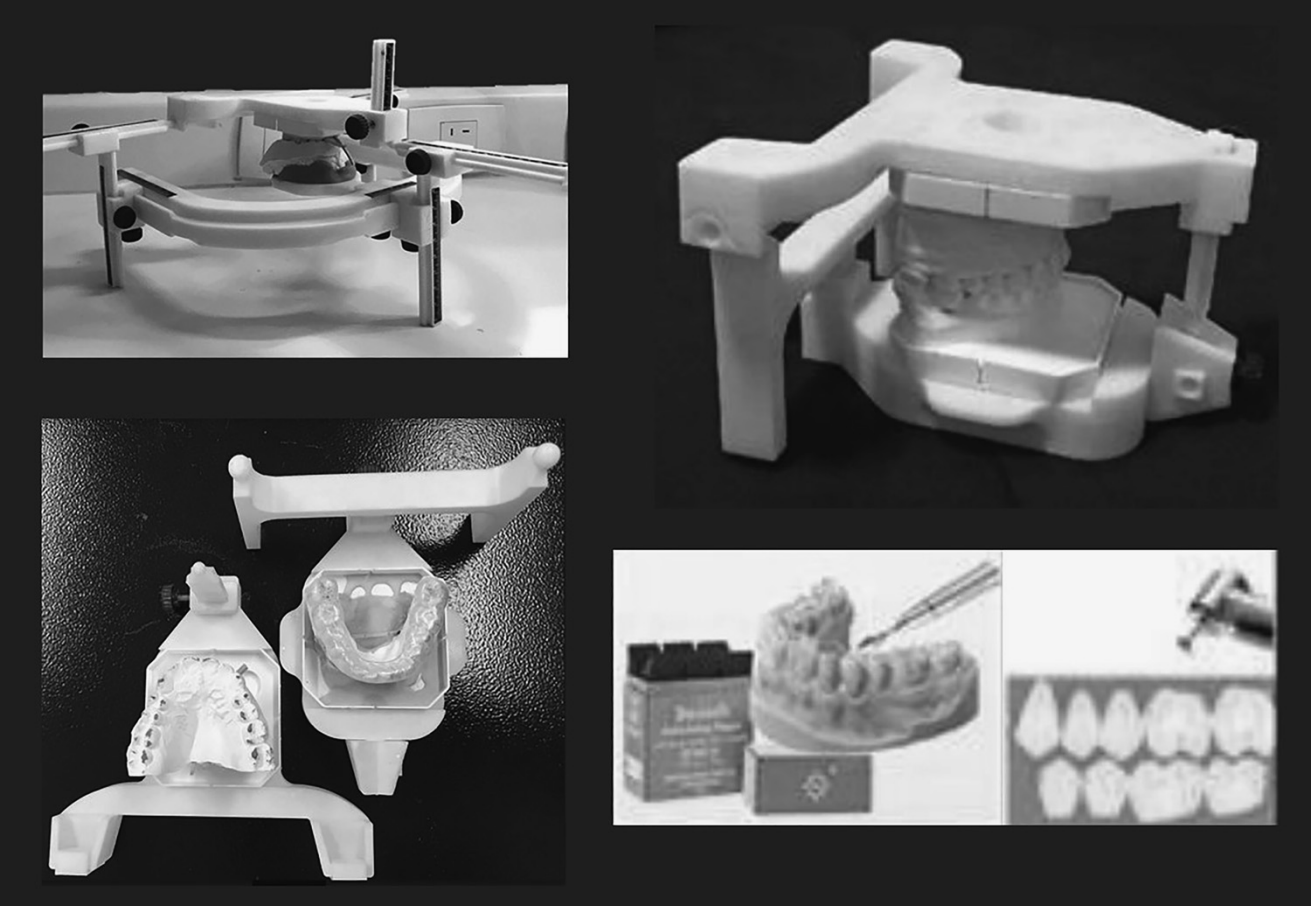
Inferior occlusal splint and selective occlusal adjustment based on the neuro-occlusal rehabilitation constructed with high-technology gnathostatic device model that uses Camper's plane for diagnosis.

The initial therapeutic indication was the occlusal splint based on the NOR usage day and night except when eating and resting if necessary. An inferior full-coverage ERKOLOC-PRO 3MM (Erkodent Erich Kopp GMBH) occlusal splint was applied. This occlusal splint is made according to NOR principles using high technology (HT) gnathostatic device, which considers the Camper's plane when assembling the gnathostatic models. The therapeutic change posture is oriented by the gnathostatic model in the HT gnathostatic device. The disocclusion of occlusal splint is oriented by Camper's plane. According to NOR concepts, the Camper's plane as a disocclusion reference intends to achieve neuromuscular balance and functional stability, using the same principles of the oral appliance with mandibular advancement device for obstructive sleep apnea treatment. 7 9 Bausch carbon (BK 200 μ) and maxcut tungsten cutters for the handpiece were used for adjustment ( Fig. 1 ).

After 1 week of follow-up occlusal splint usage, the patient reported remission in tinnitus and cervicalgia, without TMJ pain symptoms, except after chewing. The pain decreased to ¾ in VAS. Then, dental occlusion was checked, and observed premature contacts in the left side second molars distal slopes, which received selective occlusal adjustment based on the NOR ( Fig. 1 ).

After the selective occlusal adjustment based on NOR, the pain disappeared completely, and the mouth opening had improved by 1 cm. All symptoms reached zero during 15 days, 30 days, and 60 days follow-up, except the tension in the temporal region due to bruxism, during physical activity, and when sleeping without the occlusal splint usage. Due to these complaints, we oriented her to continue occlusal splint usage during exercise and sleep for total signs and symptoms control to maintain her quality of sleep and life.

## Discussion

This report presents a clinical case of TMJ dysfunction 1 associated with tinnitus, 2 bruxism, 4 5 and cervicalgia. 3 This rare association, especially considering the overall treatment with an analogic occlusal splint and selective occlusal adjustment based on NOR, improved all symptoms, probably improving occlusion balance. The synergy of oral–cervical muscles and fascia chains helped body awareness and posture. 10

Although the exact physiological effects of occlusal splint on TMJ dysfunction treatment are still unclear, with difficulties in determining the clinical effectiveness, some studies showed that occlusal splints decrease pain and improve cervical mobility in cases of TMJ dysfunction and bruxism. Although there is a lack of evidence of occlusion role in the etiology of TMJ dysfunction and bruxism, the occlusal relationship cannot be ignored between mandible posture, cranial base, cervical spine, TMJ dysfunction, and neck pain. 10 The dental occlusion biomechanics is a critical aspect of clinical dentistry, and stomatognathic function knowledge allows a subtle recognition between dental occlusion and TMJ dysfunctions. 11 In addition, a careful individual analysis of occlusal dynamics was required to determine its causes and effects in this challenging case report. Thus, we included the complementary NOR diagnostic to therapy.

The improvement in muscle TMJ dysfunction and tinnitus was relieved due to occlusal splint usage and selective occlusal adjustment based on the NOR approach, improving the oral–cervical posture with an occlusal balance. It seems that occlusal interferences were a determining factor in causing the signs and symptoms in this case. Furthermore, in the first week, sleep bruxism was controlled with occlusal splint usage improving sleep quality, and teeth moving forward had an apparent reduction sensation. Concomitantly, after selective occlusal adjustment based on the NOR, there was remission of the painful symptomatology of chewing. The patient reported a feeling of occlusal comfort and postural balance.

The importance of a multidisciplinary team involving physicians, dentists, physiotherapists, and other health professionals is necessary for early diagnosis and treatment. 1 10

Although the occlusal splint was constructed by the analog method, digital technology can be used to occlusal splint construction. Functional balance was also found in a study that used digital technology adding data to the occlusal balance results. 12 In addition, using digital technologies allows the creation of a variety of occlusal splints by the individual needs. 13

We understand the limitations to demonstrating the efficacy of occlusal splint usage and selective occlusal adjustment based on the NOR in just one case. Still, we showed a challenging case report with therapy that treated and controlled all symptom disorders.

As the stomatognathic system demonstrates a remarkable ability to adapt to both structural and functional changes, this reported case shows the following possibilities: (1) to verify the causes and define the effects of different disorders and their associations; (2) to combine diagnosis and therapy based on the NOR; (3) to change patterns seeking the functional balance and general well-being.
